# Pain management in hospitalized infants: recommendations for achieving the Sustainable Development Goals

**DOI:** 10.1590/0034-7167-2023-0421

**Published:** 2025-01-10

**Authors:** Danton Matheus de Souza, Caroline Knoner Monteiro, Lisabelle Mariano Rossato

**Affiliations:** I Universidade de São Paulo. São Paulo, São Paulo, Brazil

**Keywords:** Neonatology, Pain Management, Intensive Care Units, Sustainable Development, Neonatal Nursing, Neonatología, Manejo del Dolor, Unidades de Cuidados Intensivos, Desarrollo Sostenible, Enfermería Neonatal

## Abstract

**Objective:**

to assess pain management in infants in a Neonatal Intensive Care Unit (NICU) and discuss its articulation with the Sustainable Development Goals, with a focus on promoting neonatal well-being.

**Method:**

a documentary study, retrospective in nature and quantitative approach, conducted in a NICU of a public hospital in Paraná, Brazil, between January and July 2022, with 386 medical records of infants, hospitalized for more than 24 hours, between 2019 and 2021. Data were subjected to descriptive and inferential analysis, considering p-value<0.05 as a statistical difference. National ethical guidelines were respected.

**Results:**

all infants underwent at least one painful procedure, but only 13.7% had documented pain. Pharmacological interventions, such as fentanyl (25.9%), and non-pharmacological interventions, such as breastfeeding encouragement (86%) were used. Only 2.8% were reassessed.

**Conclusion:**

there was a devaluation of neonatal pain management that may perpetuate neonatal well-being and sustainable development.

## RESUMEN

**Objetivo:**

evaluar el manejo del dolor en recién nacidos en la Unidad de Cuidados Intensivos Neonatales (UCIN) y discutir su conexión con los Objetivos de Desarrollo Sostenible, con enfoque en la promoción del bienestar neonatal.

**Método:**

estudio documental, de carácter retrospectivo y enfoque cuantitativo, realizado en una UCIN de un hospital público de Paraná, Brasil, entre enero y julio de 2022, con 386 prontuarios de recién nacidos hospitalizados por más de 24 horas, entre 2019 y 2021. Los datos fueron sometidos a análisis descriptivo e inferencial, considerando como diferencia estadística el valor de p<0,05. Se respetaron las directrices éticas nacionales.

**Resultados:**

todos los neonatos fueron sometidos a al menos un procedimiento doloroso, pero sólo el 13,7% tuvo dolor documentado. Hubo uso de intervenciones farmacológicas, como el fentanilo (25,9%), y no farmacológicas, como el fomento de la lactancia materna (86%). Sólo el 2,8% fueron reevaluados.

**Conclusión:**

se observó una devaluación del manejo del dolor neonatal, que puede perpetuar el bienestar neonatal y el desarrollo sostenible.

## INTRODUCTION

Aiming to create conditions for sustainable, inclusive and economic growth, the United Nations established 17 Sustainable Development Goals (SDGs), with 169 targets, to be achieved by 2030^(1)^. Sustainable development can be defined as growth that meets the needs of the present without compromising future generations^(2)^. In this context, health is specifically indicated in the third objective, with the promotion of general well-being, and is influenced by all the others, considering them as social determinants^(1,2)^. When setting health goals for the future, special attention should be given to infants (zero to 28 days), a critical stage during an individual’s development^(3)^.

In the 3^rd^ SDG, the goal is to reduce neonatal mortality^(1)^. This has permeated technological advances (hard technologies, such as incubators and vaccines), allowing an increase in infant survival. In a systematic review that aimed to analyze the causes of deaths of children under 5 years old between 2000 and 2019 in 194 countries, a reduction in infant deaths was observed^(3)^. In contrast, survivors remain in Neonatal Intensive Care Units (NICUs) for a long time, marking a life of countless challenges^(4)^, making it necessary to reflect on this context in search of their well-being.

The NICU exposes infants to numerous stimuli that can be detrimental to their development, such as the experience of pain^(4,5)^. Pain can be defined as an unpleasant sensory and emotional experience associated with, or similar to, actual or potential tissue injury^(6)^; this definition includes manipulations, pathology and all the emotional burden that can come with hospitalization, and when not alleviated, it can be an obstacle to achieving the SDGs. In the development of world science, for many years, it was believed that infants did not feel pain. However, since the mid-1980s, the literature indicates that the neural pain pathway development is present at the 24^th^ week of gestational age and that, unlike children, infants have refined nociceptive inhibitory mechanisms to reduce pain, leading to a systemic repercussion experienced for a longer time^(7,8)^.

Infants, especially premature babies, experience a critical moment for neurodevelopment, requiring protective actions^(9)^. Pain can impact this process and lead to long-term repercussions, as seen in a prospective cohort that followed more than 100 premature infants (between 24 and 32 weeks of gestational age) until they were 8 years old, demonstrating that neonatal pain stress was associated with internalizing behaviors throughout child development^(10)^. In addition to the above, it is indicated that pain can lead to clinical changes, behavioral dysregulation, sensory processing problems and poor executive functioning^(8,11)^.

The experience of pain in infants occurs mainly through painful procedures. A systematic review of 18 observational studies indicated that infants experience an average of between seven and 17 painful and stressful procedures per day, with more than 6,000 procedures per hospitalization^(12)^, high and worrying number. In Brazil, there is underreporting of neonatal pain with low estimates^(8)^.

Moving towards improving NICU practices, the Brazilian National Policy for Comprehensive Child Healthcare (PNAISC - *Política Nacional de Atenção Integral à Saúde da Criança*) has two strategic axes that should guide care. The first refers to humanized and qualified care, from pregnancy to newborn care. The second reinforces the importance of breastfeeding and healthy eating^(13)^. These two aspects are intrinsically connected to the SDGs and pain relief. In this context, the aforementioned context demonstrates that reflecting on the experience of pain in association with the SDGs is essential to achieve sustainable growth, with humanized and qualified care.

Scientific progress has been made in recent years in neonatal pain management, with the dissemination of validated scales for qualified assessment, a greater number of studies on drugs and non-pharmacological interventions^(6-8,14)^. However, the assessment of the translation of this literature into clinical practice is still timid, which led to the research question of this study: how is pain management performed in infants hospitalized in a Brazilian NICU and what are its interfaces with the SDGs, with a focus on promoting neonatal well-being? Continuing research on this topic is necessary and indicated in the SDG targets as a means for implementing good practices^(1)^, in addition to one of the essential competencies of neonatal nurses^(15)^.

## OBJECTIVE

To assess pain management in infants in the NICU and discuss its relationship with the SDGs, with a focus on promoting neonatal well-being.

## METHODS

### Ethical aspects

The study received ethical approval from the *Universidade de São Paulo* School of Nursing. Due to the retrospective approach, consent was waived, with the main researcher signing the Term of Responsibility. The ethical guidelines of Resolution 466/12 of the Brazilian National Health Council were respected.

### Study design

This is a documentary study of a retrospective nature and a quantitative approach. To guide its methodology, STrengthening the Reporting of OBservational studies in Epidemiology (STROBE) was used^(16)^.

### Study place, period and population

The study was conducted in a NICU of a public tertiary teaching hospital in Curitiba, Paraná, Brazil. This NICU has 20 beds, with ten intensive care beds, eight semi-intensive beds and two for Kangaroo Mother Care, with hospitalization of extremely premature or full-term infants, with congenital malformations and/or pathological alterations. Data collection took place between January and July 2022, with medical records of infants hospitalized between 2019 and 2021.

### Eligibility criteria

Medical records of premature and full-term infants (zero to 28 days of corrected age) hospitalized in the NICU for more than 24 hours between 2019 and 2021 were included. Medical records that were unavailable due to use in consultations or by other researchers, in addition to incomplete records, were excluded.

### Sample calculation

Sample size was determined to assess pain management in neonatology, with a 50% proportion and a 5% margin of error. Thus, it was indicated that at least 386 medical records should be collected, with a 95% confidence level.

### Data collection

The co-participating institution receives an average of 3,000 births per year, and the NICU receives an annual average of 200 infants. Therefore, it was decided to recruit participants using the simple random sampling technique. After ethical approval, a list of hospitalizations during the indicated period was given to the researcher, and a draw (mediated by an external person) was carried out to collect 386 medical records. Medical records were requested from the institution’s archives department, and were delivered and read in full and in detail. Collection was conducted by a neonatology nurse, a master’s student in science, who was previously trained by the main researcher. For collection, an instrument was formulated with variables characterizing infants, hospitalization context, painful procedures, invasive devices and pain management.

It should be noted that painful procedures were considered, such as invasive ones, which cause damage to the skin, mucosa, or when there is removal or introduction of foreign material into the airways, digestive or urinary tract^(12)^, pain management, with the stages of assessment, intervention and reassessment, and non-pharmacological interventions, all documented practices for pain prevention or relief^(8)^. The institution does not have a pain management protocol, only the recommendation of assessment using the Neonatal Infant Pain Scale (NIPS). If the researcher did not find a record on the scale, the pain record and parameter used for assessment were searched in the notes.

### Data analysis

Data were entered into spreadsheets in Microsoft Excel^®^ without characterizing the child. The database was stored in a cloud owned by the researchers. A descriptive analysis was conducted with simple and relative frequencies, estimates of mean and standard deviation, and an inferential analysis, with association of variables, using the chi-square test, in the R 4.0.4 software. A p-value <0.05 (5%) was considered a statistical difference in a 95% Confidence Interval. To discuss the findings, the data were linked to the SDGs, with a focus on the 3^rd^ SDG (Ensure healthy lives and promote well-being for all at all ages)^(1)^.

## RESULTS

The sample of this study was 386 infants. There was a prevalence of male infants (56.2%), premature infants younger than 37 weeks (68%), born by cesarean section (55.4%), with adequate weight for gestational age (75.2%), hospitalized in the NICU due to conditions originating in the neonatal period (90.4%), with an average of 25 days, using non-invasive ventilation (53.1%). Moreover, 8.3% had a congenital malformation, and 1.9% died (Table 1).

**Table 1:** Characterization of participating infants (N=386). Curitiba, Paraná, Brazil, 2023

Variable	n (%)	95% CI
Sex		
Female	169 (43.8)	38.92-48.77
Male	217 (56.2)	51.23-61.08
Gestational age		
Extremely preterm (<28 weeks)	25 (6.9)	4.43-9.39
Very preterm (between 28 and 32 incomplete weeks)	56 (14.5)	11.34-18.38
Moderately preterm (between 32 and 34 incomplete weeks)	83 (21.5)	17.7-25.87
Late preterm (between 34 and 37 incomplete weeks)	97 (25.1)	21.06-29.69
Full term (>37 weeks)	125 (32.0)	27.91-31.21
Type of childbirth		
Normal	172 (44.6)	38.89-53.28
Cesarean section	214 (55.4)	50.45-60.32
Weight classification		
Appropriate for gestational age (<10th percentile)	290 (75.2)	70.58-79.18
Large for gestational age (10th to 89th percentile)	30 (7.7)	5.5-10.88
Small for gestational age (>90th percentile)	66 (17.1)	13.67-21.17
Congenital malformation	32 (8.3)	5.93-11.47
Clinical diagnosis		
Conditions originating in the neonatal period^[1]^	349 (90.4)	87.07-92.97
Infectious or parasitic diseases^[2]^	8 (2.0)	1.05-4.04
Congenital, genetic alterations or deficiencies[3]	18 (4.6)	2.97-7.25
Social cases^[4]^	11 (2.8)	1.6-5.03
Ventilatory device		
Ambient air	19 (4.9)	3.17-7.56
Nasal catheter	163 (42.2)	37.4-47.21
Hood box	146 (37.8)	33.13-42.76
Non-invasive mechanical ventilation	205 (53.1)	48.12-58.03
Mechanical ventilation	144 (37.3)	32.63-42.23
Hospitalization classification		
Non-prolonged (<30 days)	260 (67.4)	62.53-71.84
Prolonged (>30 days)	126 (32.6)	28.16-37.47
Death	7 (1.9)	1.41-4.7
Prenatal care	293 (75.9)	71.4-79.9
	Mean (+SD)	Min-max
Number of prenatal consultations	7 (3.45)	0-20
Days of hospitalization	25.5 (24.6)	1-193
Maternal age	26.6 (7.64)	8-77

*Note: [1] Conditions originating in the neonatal period - prematurity, respiratory distress, early and late neonatal infection, hyaline membrane, abdominal distension, meconium aspiration syndrome, enterocolitis, neonatal jaundice, asphyxia, cyanosis, pneumothorax, pulmonary hypertension, fetal distress, fetal centralization, seizures and the like; [2] Infectious or parasitic diseases - exposure to B24, exposure to syphilis, exposure to toxoplasmosis, COVID+ mother and COVID+ newborn; [3] Congenital, genetic alterations or deficiencies - heart disease, cleft lip and palate, achondroplasia, esophageal atresia, diaphragmatic hernia, brain malformation, Edwards syndrome, Vater syndrome, trisomy 21, congenital constriction band syndrome, brain malformations; and [4] Social issues - social risk, social case, birth without prenatal care, home birth, breastfeeding difficulties; SD - standard deviation; CI - Confidence Interval; Min-max - minimum-maximum.*

All infants underwent a painful procedure for collecting samples or inserting devices. Arterial, calcaneal and venous punctures (for peripheral venous access) were the most common, with up to 70 procedures performed (arterial punctures) per infant during their hospitalization. It is important to note that the number of procedures performed was not documented, which limited a more in-depth analysis (Table 2).

**Table 2:** Painful devices and procedures (N=386). Curitiba, Paraná, Brazil, 2023

Variables	n (%)	Mean (+SD)	Min-max	95% CI
Peripheral venous access	357 (92.5)	4.85 (4.0)	0-32	89.42-94.72
Umbilical arterial catheterization	148 (38.3)	0.37 (0.65)	0-3	33.63-43.28
Peripherally inserted central catheter	114 (29.5)	-	-	25.2-34.27
Phlebotomy	16 (4.2)	0.06 (0.35)	0-4	2.57-6.63
Oro/nasogastric catheter	364 (94.3)	-	-	91.52-96.21
Oro/nasoenteral catheter	9 (2.3)	-	-	1.23-4.37
Indwelling urinary catheter	21 (5.4)	-	-	3.59-8.17
Lumbar puncture	98 (25.4)	0.41 (0.85)	0-6	21.3-29.96
Heel puncture	386 (100)	-	-	99.01-100
Arterial puncture	486 (100)	9.18 (8.19)	0-70	99.01-100

*Note: SD - standard deviation; CI - Confidence Interval; Min-max - minimum-maximum.*

No infant was assessed for pain using scales, with subjective assessment parameters being documented, such as pain face (8.3%). Thus, 13.7% presented pain, which was treated through pharmacological interventions, such as continuous or intermittent fentanyl (25.9%), or non-pharmacological interventions, such as encouraging breastfeeding (86%), skin-to-skin contact/Kangaroo Mother Care (66.9%) and concentrated care/minimal touch (42.7%). It is noteworthy that only 2.8% had a documented pain reassessment (Table 3). Regarding non-pharmacological interventions, the professionals involved in their documentation/prescription were nurses (34.4%), physicians (28.7%) and physiotherapists (18.9%).

**Table 3:** Neonatal pain management (N=386). Curitiba, Paraná, Brazil, 2023

Variables	n (%)	95% CI
Pain assessment^*^		
Recording pain in the medical record	53 (13.7)	10.65-17.52
Pain assessment using a scale	0	0
Crying (pain assessment parameter)	23 (5.9)	4-8.78
Agitation (pain assessment parameter)	14 (3.6)	2.17-6
Pain face (pain assessment parameter)	32 (8.29)	5.93-11.47
Pharmacological intervention^*^		
Use of paracetamol/dipyrone	28 (7.2)	5.07-10.28
Use of continuous/intermittent fentanyl	100 (25.9)	21.79-30.5
Use of continuous/intermittent midazolam	21 (5.4)	3.59-8.17
Non-pharmacological intervention^*^		
Reduced light	132 (34.2)	29.64-39.06
Focused care/minimal touch	165 (42.7)	37.91-47.73
Breastfeeding encouragement	332 (86.0)	82.19-89.12
Skin-to-skin contact/Kangaroo Mother Care	257 (66.9)	61.73-71.1
Non-nutritive sucking	35 (9.0)	6.59-12.35
Sweetened solution	4 (1.0)	0.4-2.63
Facilitated restraint	8 (2.0)	1.05-4.04
Shantala massage	4 (1.0)	0.4-2.63
Pain reassessment	11 (2.8)	1.6-5.03

Note:

* More than one parameter for each infant; CI - Confidence Interval.

**Figure 1:**
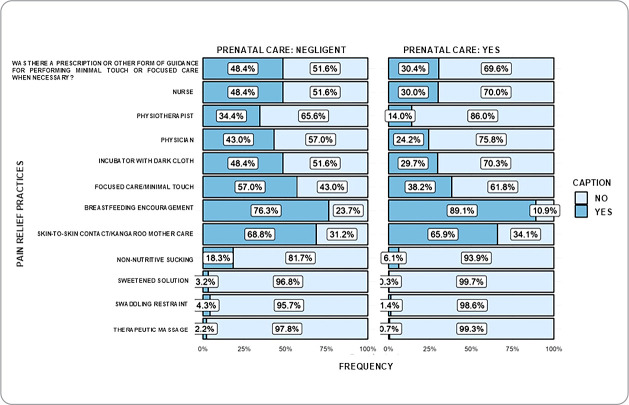
Documentation of non-pharmacological interventions and their association with prenatal care. Curitiba, Paraná, Brazil, 2023

**Figure 2:**
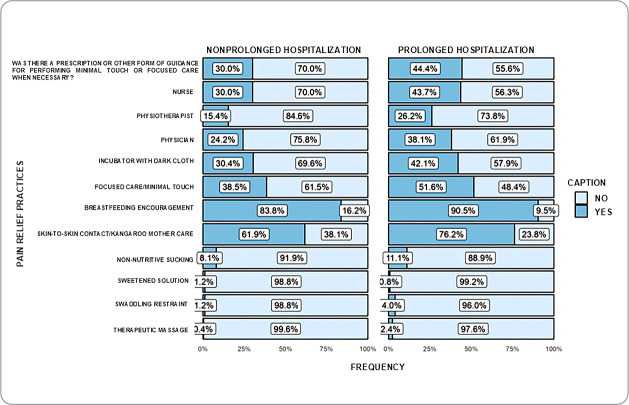
Documentation of non-pharmacological interventions and their association with length of hospital stay. Curitiba, Paraná, Brazil, 2023

From the association of variables, it was observed that prenatal care was associated with the use of breastfeeding encouragement (p<0.05), while negligent prenatal care (<3 consultations) was associated with the use of non-nutritive sucking, sweetened solution, swaddling restraint and therapeutic massage (p<0.05) (Figure 1). Length of hospitalization >30 days was associated with the documentation of non-pharmacological interventions, such as reduced light, concentrated care/minimal touch, skin-to-skin contact/Kangaroo Mother Care (p<0.05) (Figure 2).

## DISCUSSION

In this study, in the total sample, a reduced number of neonatal deaths (1.9%) was observed, in line with the target of reducing neonatal mortality of the 3^rd^ SDG^(1)^. The causes of hospitalization support the literature and highlight the importance of studies with infants, since complications of prematurity and labor are indicated as two of the main causes of death among children under 5 years old^(3)^, requiring a longitudinal approach. In a movement towards the population’s well-being^(1)^, infants deserve special attention.

As mentioned above, pain management can be integrated into the SDGs. Management begins at the assessment stage, which is crucial for identifying care needs and quickly conducting interventions^(14)^. Here, only 53 infants had documented pain associated with painful procedures, without assessment by scales (although NIPS was available), a worrying fact that may be associated with professional knowledge, beliefs and attitudes. In a study with 51 NICU nurses, it was observed that 70.6% were not aware of scales for pain assessment (knowledge); 8% disagreed that infants felt pain; 6% disagreed that pain could have long-term repercussions (beliefs); and 11.8% reported that they did not record pain management in their medical records (attitudes)^(17)^. This supports another investigation, with 256 healthcare professionals from neonatal units, which demonstrated physicians’ and nurses’ low knowledge about the stages of pain management^(18)^.

The aforementioned aspect can be translated to other care scenarios, such as this study, and may have influenced the low number of pain documentations, despite estimating that pain may have been experienced by all infants in the total sample. The number of reassessments is worrying, as only 2.8% of infants had new documentation. This reiterates the importance of addressing professional knowledge, beliefs and attitudes in future studies, in line with the 4^th^ SDG, which aims to ensure quality education for all individuals, also integrating professionals who need to continue improving themselves in search of qualified and sustainable assistance^(1,2)^.

It is considered that all infants using mechanical ventilation or with assessed pain should receive analgesics^(7,10)^. Despite the high number of pharmacological interventions in this study (Table 3), there is still a long way to go. Professionals are concerned about using medications in infants, as hepatic metabolism and glomerular filtration are reduced^(6,10)^, which opens doors for the growth of non-pharmacological interventions that, in this public, unlike children, can have them as an adjuvant for pain relief^(15)^.

Given neonatal nurses’ essential skills, the identification of alternative solutions for promoting infant health is encouraged^(15)^ and, in addition to the 3^rd^ SDG, in search of promoting well-being^(1)^, non-pharmacological interventions should be highlighted. These have mechanisms of action that focus on preventing disorganization, with neurological and endocrine effects, and their use is recommended between five and ten minutes before and/or during painful procedures^(6,7)^. However, in this study, there is little documentation of these interventions, especially the most widespread ones, such as sweetened solution^(15)^. This aspect may be associated with two points: fear or professional routine.

In a cross-sectional study with healthcare professionals from neonatal units, it was noted that physicians and nurses report fears about using non-pharmacological interventions in clinical practice, fearing repercussions on infants’ health, considering their clinical lability^(12)^. It is reiterated that this fear is not something negative, but scientific empowerment is important for the transition from fear to safety (3^rd^ SDG). Non-pharmacological interventions are not risk-free and should be used appropriately, such as suction measures, which should not be used in infants with neurological abnormalities and transient choking (non-nutritive), and gastrointestinal tract abnormalities (nutritive)^(8)^.

Another point may be linked to the professional routine, which focuses on providing a neutral environment, free from stimuli, to preserve oxygen consumption, body temperature and metabolism, with pain management and its documentation taking a back seat^(9)^. Here, there were 132 records of reduced luminosity, an environmental intervention that corroborates the above. Breastfeeding documentation may be high, considering the institutional policies of baby-friendly hospitals, which aim to encourage breastfeeding throughout infant hospitalization, in line with the 2^nd^ SDG^(1)^ and the second axis of PNAISC^(13)^.

In this study, it was observed that the number of painful procedures documented was high, such as 70 arterial punctures, 32 venous accesses, six lumbar punctures and four phlebotomies. This estimate may be an underestimate, and professionals often document only the success of the procedure, not the number of failed attempts. In venipunctures, for instance, the number of attempts can be high, considering the developing venous network. Another point is that these procedures are of vital importance for maintenance of infants’ life. Exposing the number of attempts is not a criticism of their performance, but rather the need to reflect on strategies so that infants have a reduction in suffering during the procedure, moving, once again, on the path towards neonatal well-being (3^rd^ SDG)^(1)^.

There are other routine care procedures that, despite going against the definition of a painful procedure^(12)^, can cause pain if not properly conducted, such as hygiene care, anthropometric assessment and neonatal screening, which should be reflected in future studies.

It is urgent to disseminate non-pharmacological interventions to professionals, as recommended in the 17^th^ SDG (diffusion of technologies). However, it is urgent that interventions be named as such, moving towards a culture where they are seen and valued as a care technology, just like hard technologies, and move from options to professional obligations, integrating protocols that aim at well-being in the NICU. In this study, eight non-pharmacological interventions (with proven effectiveness in the literature) are observed^(6,19-22)^. However, a recent Brazilian guideline recognized only suction (nutritive, non-nutritive and with sweetened solutions), skin-to-skin contact and facilitated restraint^(8)^, demonstrating that this reflection should also follow the scientific literature.

Among the numerous associations conducted, prenatal care and length of hospital stay were associated with the use of non-pharmacological interventions. As for prenatal care, it is noted that 24.1% of mothers did not attend it (negligent prenatal care), a worrying fact that goes against the recommendations of the 5^th^ SDG (women’s empowerment)^(1)^. This low number may be associated with the COVID-19 pandemic, which imposed contextual changes on the health system, with the low number of consultations during the period already recognized in the literature^(23)^. Furthermore, quality prenatal care is an initial encouragement for positive parenting^(10)^, and in this study, its presence was associated with breastfeeding encouragement, which can be associated with greater guidance and maternal preparation during this monitoring (2^nd^ and 5^th^ SDG)^(1)^.

Negligent prenatal care was associated with the use of non-pharmacological interventions, a positive aspect. One hypothesis for this finding is that this variable may have encouraged the professional to conduct interventions aimed at infant well-being (3^rd^ SDG)^(1)^. Another possibility is for the co-participating hospital to be a baby-friendly hospital, which integrates Kangaroo Mother Care and breastfeeding as integral parts of care, ensuring that infants are connected to the two axes of PNAISC^(13)^. These interventions were the most prevalent in pain relief, a result that may corroborate the aforementioned aspect.

There are non-pharmacological interventions that depend on family collaboration, such as encouraging breastfeeding, skin-to-skin contact/Kangaroo Mother Care and therapeutic massage (where it is recommended that the family conducts it, supervised by professionals)^(12,22,24)^. Its implementation can be a predictor of the bond between the dyad, providing not only neonatal well-being, but also maternal well-being (3^rd^ and 5^th^ SDGs)^(1)^. The NICU can be an environment that imposes numerous challenges to women’s health, in addition to hormonal changes after childbirth, which can harm the bond^(9)^.

The bond between the dyad is crucial for infants’ neurophysiological, physical and psycho-emotional development. However, the COVID-19 pandemic influenced this process, as seen in a cross-sectional study with 127 dyads, in which it was observed that unplanned pregnancy and probable maternal mental health problems harmed the bond^(23)^. The 3^rd^ and 5^th^ SDGs are once again integrated into this process, requiring a look at women’s health in the context of the NICU^(1)^. Despite the above, the number of skin-to-skin contact/Kangaroo Mother Care sessions in this study was high. However, it is important to note that all infants should receive this, as, in addition to providing pain relief, it is an encouragement for positive parenting, bonding, promoting infants’ well-being and a low-cost, sustainable intervention that should be encouraged in the NICU, in accordance with humanized care, the axis of PNAISC, and with the 3^rd^ SDG^(1,13,25)^.

During the period of neurobiological vulnerability, infants may experience prolonged hospitalizations in the NICU^(10)^, and this study demonstrated the association of this variable with the use of non-pharmacological interventions. The researchers indicate as a hypothesis for this association the increased chance of, at least, documentation of the interventions, considering that this infant will be exposed to a greater number of painful procedures and a professional can make a documentation. On the other hand, it is worth considering that, in clinical practice, as the length of hospitalization increases, infants refine their nociceptors and pain organization mechanisms^(18)^, and this can cause the professional to fall into complacency in mechanical care: “We do this procedure every day”^(26)^, and it is important to emphasize that promoting well-being is an ongoing process that should guide the entire hospitalization^(1)^.

An important point is that non-pharmacological interventions were documented by nurses, physicians and physiotherapists, which demonstrates that education (4^th^ SDG) must be interdisciplinary, including all professionals who perform painful procedures^(1,7)^. Another point is that this study was conducted in Brazil; in other countries, with different cultures, interventions may have different representation, especially non-pharmacological ones, and different availability. Culturally responsive care becomes a philosophy that should also guide pain management, respecting the context in which it is inserted, a goal of the 1^st^ and 10^th^ SDGs^(1)^.

Reflecting on pain management in NICUs with the SDGs is a step towards preventing long-term impacts on maternal and child well-being. Although the study focuses on one stage of an individual’s life, it is necessary to go further, since experiences are cumulative. The experience of unrelieved pain can lead to a cascade effect that will harm children, adolescents, adults and future older adults^(2,8)^. Pain management in infants is a care for the present and the future, being in full agreement with the premise of the 2030 Agenda for Sustainable Development^(1)^.

After hospitalization, this infant will be cared for in a Healthcare Network, which must be interconnected with actions in a territory, to promote the full development of individuals. It is necessary to coordinate actions that respect the social context (1^st^ and 10^th^ SDG) and promote food security (2^nd^ SDG), quality education (4^th^ SDG), basic sanitation and respect for the environment (6^th^, 13^th^, 14^th^, 15^th^ SDG), access to sustainable technologies (7^th^, 8^th^, 9^th^, 11^th^ SDG) and universal access to healthcare services (16^th^ SDG), which have sustainable development as a guide to articulate care (17^th^ SDG)^(1,2)^. Pain management in the NICU is a small time frame of the importance of articulating care in search of well-being (3^rd^ SDG)^(1,2)^, however it is as important as those mentioned above.

### Study limitations

This study has limitations due to its retrospective design, which leads to a loss of information that would be essential for a more in-depth analysis of the phenomenon, such as the number of painful procedures performed, including attempts, and assessment of pain performed using subjective parameters, which may have underestimated the actual pain experience.

### Contributions to health, nursing or public policy

In a world that is moving towards sustainable development of individuals, neonatal pain management becomes crucial for healthcare professionals, with emphasis on nurses who work in pain management. This study demonstrated a high number of painful procedures, but with a low number of documentation of pain and interventions. This aspect may represent a clinical practice that needs to be redefined, moving towards the integration of the SDGs in safe and quality care for infant health, reducing the repercussions of unrelieved pain in the long term. The data from this study and the reflections discussed may be a starting point for this long journey.

## CONCLUSION

In this study, it was observed that pain management in infants was undervalued. All participants were exposed to at least one painful procedure, however only 13.7% had pain documentation, without the use of a validated scale, although it was available. There was use of pharmacological interventions, such as continuous or intermittent fentanyl, and non-pharmacological interventions, with breastfeeding encouragement and Kangaroo Mother Care prevailing, with these interventions associated with prenatal care and prolonged hospitalization (p<0.05). Only 2.8% of infants were reassessed. This context is linked to the SDGs, with a focus on well-being, considering that the experience of pain can have repercussions throughout development, and it is important that pain management is integrated with the goals recommended for sustainable development.
