# Supplementary material for: Pain management in hospitalized infants: recommendations for achieving the Sustainable Development Goals
Source: Rev Bras Enferm. 2025 Jan 10;77(Suppl 2):e20230421. doi: 10.1590/0034-7167-2023-0421 (PMC11726822; doi:10.1590/0034-7167-2023-0421)
Supplement: Translation

# Translation: Manejo da dor em neonatos hospitalizados: recomendações para alcançar os Objetivos de Desenvolvimento Sustentável

**DOI:** 10.1590/0034-7167-2023-0421pt

**Authors:** Danton Matheus de Souza, Caroline Knoner Monteiro, Lisabelle Mariano Rossato

**Affiliations:** I Universidade de São Paulo. São Paulo, São Paulo, Brasil

**Keywords:** Neonatologia, Manejo da Dor, Unidades de Terapia Intensiva, Desenvolvimento Sustentável, Enfermagem Neonatal

## Abstract

**Objetivo:**

avaliar o manejo da dor de neonatos em Unidade de Terapia Intensiva Neonatal (UTIN) e discutir sua articulação com os Objetivos de Desenvolvimento Sustentável, com enfoque na promoção do bem-estar neonatal.

**Método:**

estudo documental, de caráter retrospectivo e abordagem quantitativa, conduzido em uma UTIN de hospital público no Paraná, Brasil, entre janeiro e julho de 2022, com 386 prontuário de neonatos, hospitalizados por mais de 24 horas, entre 2019 e 2021. Os dados foram submetidos à análise descritiva e inferencial, considerando o valor de p<0,05 como diferença estatística. Respeitaram-se as diretrizes éticas nacionais.

**Resultados:**

todos os neonatos foram submetidos a, no mínimo, um procedimento doloroso, mas apenas 13,7% tiveram dor documentada. Houve uso de intervenções farmacológicas, como fentanil (25,9%), e não farmacológicas, como estímulo à amamentação (86%). Apenas 2,8% foram reavaliados.

**Conclusão:**

observou-se uma desvalorização do manejo da dor neonatal que pode perpetuar no bem-estar neonatal e desenvolvimento sustentável.

## INTRODUÇÃO

Almejando criar condições para um crescimento sustentável, inclusivo e econômico, as Nações Unidas estabeleceram 17 Objetivos de Desenvolvimento Sustentável (ODS), com 169 metas, para serem atingidos até 2030^(1)^. O desenvolvimento sustentável pode ser definido como o crescimento que atende às necessidades do presente sem comprometer a geração futura^(2)^. Neste contexto, a saúde é indicada, especificamente, no terceiro objetivo, com a promoção do bem-estar geral, e é influenciada por todos os outros, considerando-os determinantes sociais^(1,2)^. Ao organizar metas de saúde para o futuro, atenção especial deve ser dada a neonatos (zero a 28 dias), etapa crítica durante o desenvolvimento de um indivíduo^(3)^.

No 3º ODS, é indicado como meta a redução da mortalidade neonatal^(1)^. Essa permeou o avanço tecnológico (tecnologias duras, como incubadoras e vacinas), permitindo um aumento da sobrevida de neonatos. Em revisão sistemática que visou analisar as causas de óbitos de crianças menores que 5 anos entre 2000 e 2019 em 194 países, observou-se uma redução de óbitos em neonatos^(3)^. Em contrapartida, os sobreviventes ficam em Unidades de Terapia Intensiva Neonatal (UTINs) por um tempo prolongado, marcando uma sobrevida com inúmeros desafios^(4)^, sendo necessário refletir sobre este contexto em busca do seu bem-estar.

A UTIN expõe o neonato a inúmeros estímulos que podem ser prejudiciais ao seu desenvolvimento, tal como a vivência da dor^(4,5)^. A dor pode ser definida como uma experiência sensitiva e emocional desagradável associada, ou semelhante àquela associada, a uma lesão tecidual real ou potencial^(6)^; essa definição integra as manipulações, a patologia e todo a carga emocional que pode caminhar com a hospitalização, sendo que, quando não aliviada, pode ser um impasse para atingir os ODS. No desenvolvimento da ciência mundial, por muitos anos, acreditava-se que neonatos não sentiam dor. Todavia, desde meados da década de 1980, a literatura indica que o desenvolvimento da via neural da dor está presente na 24ª semana de idade gestacional e que, diferente de crianças, os neonatos possuem mecanismos inibitórios nociceptivos em refinamento para reduzir a dor, levando a uma repercussão sistêmica vivenciada por maior tempo^(7,8)^.

Os neonatos, especialmente os prematuros, vivenciam um momento crítico para o neuro desenvolvimento, sendo necessárias ações de proteção^(9)^. A dor pode impactar esse processo e levar a repercussões a longo prazo, como visto em coorte prospectiva que acompanhou mais de 100 neonatos prematuros (entre 24 e 32 semanas de idade gestacional) até seus 8 anos, demonstrando que o estresse com a dor neonatal foi associado a comportamentos de internalização ao longo do desenvolvimento infantil^(10)^. Para além do exposto, é indicado que a dor pode levar a alterações clínicas, desregulação comportamental, problemas de processamento sensorial e um funcionamento executivo deficiente^(8,11)^.

A vivência de dor em neonatos ocorre principalmente por intermédio de procedimentos dolorosos. Revisão sistemática com 18 estudos observacionais indicou que neonatos vivenciam uma média entre sete e 17 procedimentos dolorosos e estressantes por dia, com mais de 6 mil procedimentos por hospitalização^(12)^, número elevado e preocupante. No Brasil, há uma subnotificação da dor neonatal com baixas estimativas^(8)^.

Caminhando para um aperfeiçoamento da prática em UTIN, a Política Nacional de Atenção Integral à Saúde da Criança (PNAISC) traz dois eixos estratégico que devem guiar a assistência. O primeiro se refere a uma atenção humanizada e qualificada, desde a gestação até o cuidar do recém-nascido. Já o segundo reforça a importância do aleitamento materno e alimentação saudável^(13)^. Esses dois aspectos se conectam, de forma intrínseca, aos ODS e ao alívio da dor. Nesse caminhar, todo o contexto supracitado demonstra que refletir sobre a vivência da dor em associação aos ODS é emergente para atingir um crescimento sustentável, com uma assistência humanizada e qualificada.

Avanço científico foi realizado nos últimos anos no manejo da dor neonatal, com a difusão de escalas validadas para uma avaliação qualificada, maior número de estudos sobre fármacos e intervenções não farmacológicas^(6-8,14)^. Entretanto, a avaliação da tradução dessa literatura na prática clínica ainda se mostra tímida, o que levou à pergunta de pesquisa deste estudo: como ocorre o manejo da dor em neonatos hospitalizados em UTIN brasileira e quais são suas interfaces com os ODS, com enfoque na promoção do bem-estar neonatal? A continuidade de investigações diante da temática é necessária e indicada nas metas dos ODS como um meio para a implementação de boas práticas^(1)^, somada a uma das competências essenciais de enfermeiros neonatais^(15)^.

## OBJETIVO

Avaliar o manejo da dor de neonatos em UTIN e discutir sua articulação com os ODS, com enfoque na promoção do bem-estar neonatal.

## MÉTODOS

### Aspectos éticos

O estudo recebeu aprovação ética da Escola de Enfermagem da Universidade de São Paulo. Devido à abordagem retrospectiva, houve dispensa do consentimento, com a assinatura da pesquisadora principal do Termo de Responsabilidade. Respeitaram-se as diretrizes éticas da Resolução n° 466/12 do Conselho Nacional de Saúde.

### Delineamento do estudo

Estudo documental, de caráter retrospectivo e abordagem quantitativa. Para guiar sua metodologia, utilizou-se o instrumento *STrengthening the Reporting of OBservational studies in Epidemiology* (STROBE)^(16)^.

### Local, período e população do estudo

O estudo foi conduzido em uma UTIN de hospital escola, terciário, público, em Curitiba, Paraná, Brasil. Essa UTIN conta com 20 leitos, com dez de terapia intensiva, oito de semi-intensiva e dois para a realização do Método Canguru, com hospitalização de neonatos prematuros extremos ou a termo, com má formação congênita e/ou alterações patológicas. A coleta de dados ocorreu entre janeiro e julho de 2022, com prontuários de neonatos hospitalizados entre 2019 e 2021.

### Critérios de elegibilidade

Foram incluídos prontuários de neonatos (zero até 28 dias de idade corrigida) prematuros e a termo hospitalizados na UTIN, por mais de 24 horas, entre o ano de 2019 e 2021. Foram excluídos os prontuários indisponíveis por uso em consultas ou por outros pesquisadores, além dos incompletos.

### Cálculo amostral

O dimensionamento amostral foi determinado para avaliar o manejo da dor em neonatologia, com proporção de 50% e margem de erro de 5%. Assim, indicou-se que fossem coletados, no mínimo, 386 prontuários, com nível de confiança de 95%.

### Coleta de dados

A instituição coparticipante recebe, em média, 3 mil nascimentos por ano, e a UTIN, uma média anual de 200 neonatos. Assim, optou-se por recrutar os participantes com a técnica de amostragem aleatória simples. Após a aprovação ética, foi entregue ao pesquisador uma lista de hospitalizações no período indicado, sendo realizado um sorteio (intermediado por pessoa externa) para a coleta de 386 prontuários. Os prontuários foram solicitados ao serviço de arquivos da instituição, sendo entregues e lidos na íntegra de forma minuciosa. A coleta foi conduzida por uma enfermeira neonatologista, mestranda em ciências, que foi treinada previamente pela pesquisadora principal. Para a coleta, foi formulado um instrumento com variáveis de caracterização dos neonatos, contexto de hospitalização, procedimentos dolorosos, dispositivos invasivos e manejo da dor.

Ressalta-se que foram considerados procedimentos dolorosos, como os invasivos, que causam lesão na pele, mucosa, ou quando há remoção ou introdução de material estranho em vias aéreas, trato digestivo ou urinário^(12)^, manejo da dor, com as etapas de avaliação, intervenção e reavaliação, e intervenções não farmacológicas, todas as práticas documentadas para prevenção ou alívio da dor^(8)^. A instituição não possui um protocolo de manejo da dor, apenas a recomendação da avaliação por intermédio da *Neonatal Infant Pain Scale* (NIPS). Caso o pesquisador não encontrasse registro na escala, buscavam-se em anotações o registro álgico e qual o parâmetro utilizado para avaliação.

### Análise dos dados

Os dados foram planilhados no *Microsoft Excel* ^®^ sem a caracterização do neonato. O banco foi armazenado em uma nuvem de domínio dos pesquisadores. Conduziram-se uma análise descritiva, com frequências simples e relativas, estimativas da média e desvio padrão, e uma análise inferencial, com associação das variáveis, por intermédio do Teste Qui-Quadrado, no *software* R 4.0.4. Considerou-se o valor de p<0,05 (5%) como diferença estatística, em um Intervalo de Confiança de 95%. Para a discussão dos achados, interligaram-se os dados com os ODS, com foco no 3º ODS (Assegurar vidas saudáveis e promover bem-estar para todos em todas as idades)^(1)^.

## RESULTADOS

A amostra deste estudo foi de 386 neonatos. Prevaleceram-se os neonatos do sexo masculino (56,2%), prematuros menores que 37 semanas (68%), nascidos por parto cesárea (55,4%), com peso adequado para idade gestacional (75,2%), hospitalizados na UTIN devido a afecções originadas no período neonatal (90,4%), com média de 25 dias, em uso de ventilação não invasiva (53,1%). Além disso, 8,3% possuíam uma má formação congênita, e 1,9% evoluíram à óbito (Tabela 1).

**Tabela 1:** Caracterização dos neonatos participantes (N=386). Curitiba, Paraná, Brasil, 2023

Variável	n (%)	IC 95%
Sexo		
Feminino	169 (43,8)	38,92-48,77
Masculino	217 (56,2)	51,23-61,08
Idade gestacional		
Pré-termo extremo (<28 semanas)	25 (6,9)	4,43-9,39
Muito pré-termo (entre 28 e 32 semanas incompletas)	56 (14,5)	11,34-18,38
Pré-termo moderado (entre 32 e 34 semanas incompletas)	83 (21,5)	17,7-25,87
Pré-termo tardio (entre 34 e 37 semanas incompletas)	97 (25,1)	21,06-29,69
A termo (>37 semanas)	125 (32,0)	27,91-31,21
Tipo de parto		
Normal	172 (44,6)	38,89-53,28
Cesárea	214 (55,4)	50,45-60,32
Classificação do peso		
Adequado para idade gestacional (percentil <10)	290 (75,2)	70,58-79,18
Grande para idade gestacional (percentil entre 10 e 89)	30 (7,7)	5,5-10,88
Pequeno para idade gestacional (percentil > 90)	66 (17,1)	13,67-21,17
Má formação congênita	32 (8,3)	5,93-11,47
Diagnóstico clínico		
Afecções originadas no período neonatal^[1]^	349 (90,4)	87,07-92,97
Doenças infecciosas ou parasitárias^[2]^	8 (2,0)	1,05-4,04
Alterações congênitas, genéticas ou deficiência^s[3^]	18 (4,6)	2,97-7,25
Casos sociais^[4]^	11 (2,8)	1,6-5,03
Dispositivo ventilatório		
Ar ambiente	19 (4,9)	3,17-7,56
Cateter nasal	163 (42,2)	37,4-47,21
Caixa de Hood	146 (37,8)	33,13-42,76
Ventilação não invasiva	205 (53,1)	48,12-58,03
Ventilação mecânica	144 (37,3)	32,63-42,23
Classificação da internação		
Não prolongada (<30 dias)	260 (67,4)	62,53-71,84
Prolongada (>30 dias)	126 (32,6)	28,16-37,47
Óbito	7 (1,9)	1,41-4,7
Realização do pré-natal	293 (75,9)	71,4-79,9
	Média (+DP)	Mín-máx
Número de consultas pré-natais	7 (3,45)	0-20
Dias de internação	25,5 (24,6)	1-193
Idade materna	26,6 (7,64)	8-77

*Nota: [1] Afecções originadas no período neonatal - prematuridades, distress respiratórios, infecção neonatal precoce e tardia, membrana hialina, distensão abdominal, síndrome da aspiração meconial, enterocolite, icterícia neonatal, asfixias, cianoses, pneumotórax, hipertensão pulmonar, sofrimento fetal, centralização fetal, crises convulsivas e semelhantes; [2] Doenças infecciosas ou parasitárias - exposição a B24, exposição à sífilis, exposição à toxoplasmose, mãe COVID+ e recém-nascido com COVID+; [3] Alterações congênitas, genéticas ou deficiências - cardiopatias, fenda labiopalatal, acondroplasia, atresia de esôfago, hérnia diafragmática, má formação cerebral, síndrome de Edwards, síndrome de Vater, trissomia do 21, síndrome de bandas de constrição congênita, malformações cerebrais; e [4] Questões sociais - risco social, caso social, parto sem realização de pré-natal, parto domiciliar, dificuldade de amamentação; DP - desvio padrão; IC - Intervalo de Confiança; Mín-máx - mínimo-máximo.*

Todos os neonatos foram submetidos a um procedimento doloroso para coleta de exames ou inserção de dispositivos. Prevaleceu-se a punção arterial, calcânea e venosa (para acesso venoso periférico), com até 70 realizações (punção arterial) por neonato em sua hospitalização. Ressalta-se que a quantidade de realização de muitos procedimentos não foi documentada, o que limitou uma análise mais aprofundada (Tabela 2).

**Tabela 2:** Dispositivos e procedimentos dolorosos (N=386). Curitiba, Paraná, Brasil, 2023

Variáveis	n (%)	Média (+DP)	Mín-máx	IC 95%
Acesso venoso periférico	357 (92,5)	4,85 (4,0)	0-32	89,42-94,72
Cateterismo arterial umbilical	148 (38,3)	0,37 (0,65)	0-3	33,63-43,28
Cateter central de inserção periférica	114 (29,5)	-	-	25,2-34,27
Flebotomia	16 (4,2)	0,06 (0,35)	0-4	2,57-6,63
Sonda oro/nasogástrica	364 (94,3)	-	-	91,52-96,21
Sonda oro/nasoenteral	9 (2,3)	-	-	1,23-4,37
Sonda vesical de demora	21 (5,4)	-	-	3,59-8,17
Punção lombar	98 (25,4)	0,41 (0,85)	0-6	21,3-29,96
Punção de calcâneo	386 (100)	-	-	99,01-100
Punção arterial	486 (100)	9,18 (8,19)	0-70	99,01-100

*Nota: DP - desvio padrão; IC - Intervalo de Confiança; Mín-máx - mínimo-máximo.*

Nenhum neonato foi avaliado para dor por intermédio de escalas, sendo documentados parâmetros de avaliação subjetivos, como a face de dor (8,3%). Assim, 13,7% apresentaram algia, que foi tratada por intermédio de intervenções farmacológicas, como fentanil contínuo ou intermitente (25,9%), ou intervenções não farmacológicas, como estímulo à amamentação (86%), contato pele a pele/Método Canguru (66,9%) e cuidados concentrados/toque mínimo (42,7%). Ressalta-se que apenas 2,8% tiveram uma reavaliação da dor documentada (Tabela 3). Diante das intervenções não farmacológicas, os profissionais envolvidos em sua documentação/prescrição foram enfermeiro (34,4%), médico (28,7%) e fisioterapeuta (18,9%).

**Tabela 3:** Manejo da dor neonatal (N=386). Curitiba, Paraná, Brasil, 2023

Variável	n (%)	IC 95%
Avaliação da dor^*^		
Registro de dor no prontuário	53 (13,7)	10,65-17,52
Avaliação da dor por uso de escala	0	0
Choro (parâmetro de avaliação da dor)	23 (5,9)	4-8,78
Agitação (parâmetro de avaliação da dor)	14 (3,6)	2,17-6
Face de dor (parâmetro de avaliação da dor)	32 (8,29)	5,93-11,47
Intervenção farmacológica^*^		
Uso de paracetamol/dipirona	28 (7,2)	5,07-10,28
Uso de fentanil contínuo/intermitente	100 (25,9)	21,79-30,5
Uso de midazolam contínuo/intermitente	21 (5,4)	3,59-8,17
Intervenção não farmacológica^*^		
Redução da luminosidade	132 (34,2)	29,64-39,06
Cuidados concentrados/toque mínimo	165 (42,7)	37,91-47,73
Estímulo à amamentação	332 (86,0)	82,19-89,12
Contato pele a pele/Método Canguru	257 (66,9)	61,73-71,1
Sucção não nutritiva	35 (9,0)	6,59-12,35
Solução adocicada	4 (1,0)	0,4-2,63
Contenção facilitada	8 (2,0)	1,05-4,04
Massagem *Shantala*	4 (1,0)	0,4-2,63
Reavaliação da dor	11 (2,8)	1,6-5,03

Nota:

* Mais de um parâmetro a cada neonato; IC - Intervalo de Confiança.

A partir da associação das variáveis, observou-se que a realização do pré-natal esteve associada ao uso do estímulo à amamentação (p<0,05), enquanto que o pré-natal negligente (<3 consultas) esteve associado ao uso de sucção não nutritiva, solução adocicada, contenção tipo enrolamento e massagem terapêutica (p<0,05) (Figura 1). Já o tempo de hospitalização >30 dias foi associado à documentação de intervenções não farmacológicas, como redução da luminosidade, cuidados concentrados/toque mínimo, contato pele a pele/Método Canguru (p<0,05) (Figura 2).

**Figura 1:** Documentação de intervenções não farmacológicas e sua associação ao pré-natal. Curitiba, Paraná, Brasil, 2023

**Figura 2:** Documentação de intervenções não farmacológicas e sua associação ao tempo de hospitalização. Curitiba, Paraná, Brasil, 2023

## DISCUSSÃO

Neste estudo, na amostra total, observou-se um número reduzido de óbitos neonatais (1,9%) condizente com a meta de redução da mortalidade neonatal do 3º ODS^(1)^. As causas de hospitalização corroboram a literatura e ressaltam a importância de estudos com neonatos, uma vez que as complicações da prematuridade e do trabalho de parto são indicadas como duas das principais causas de óbitos entre crianças menores que 5 anos^(3)^, sendo necessário um olhar longitudinal. Em um movimento em busca do bem-estar da população^(1)^, os neonatos merecem atenção especial.

Como supracitado, o manejo da dor pode ser integrado aos ODS. O manejo se inicia na etapa de avaliação, crucial para a identificação de necessidades de cuidado e, de forma ágil, conduzir intervenções^(14)^. Aqui, apenas 53 neonatos tiveram dor documentada associada a procedimento doloroso, sem intermédio de avaliação por escalas (apesar da NIPS estar disponível), fato preocupante e que pode estar associado aos conhecimentos, crenças e atitudes profissionais. Em estudo com 51 enfermeiros de UTIN, observou-se que 70,6% não conheciam escalas para avaliação da dor (conhecimento); 8% discordavam que neonatos sentiam dor; 6% discordavam que a dor poderia repercutir a longo prazo (crenças); e 11,8% referiram que não registravam o manejo da dor em prontuário (atitudes)^(17)^. Isso corrobora outra investigação, com 256 profissionais de saúde de unidades neonatais, que demonstrou o baixo conhecimento de médicos e enfermeiros sobre as etapas do manejo da dor^(18)^.

O aspecto citado pode ser traduzido a outros cenários de assistência, como o deste estudo, podendo ter influenciado o baixo número de documentações de dor, apesar de estimar que a dor pode ter sido vivenciada por todos os neonatos da amostra total. O número de reavaliações é preocupante, pois apenas 2,8% dos neonatos tiveram uma nova documentação. Isso reitera a importância da abordagem do conhecimento, crenças e atitudes profissionais em estudos futuros, indo de acordo com o 4º ODS, que visa assegurar educação de qualidade a todos os indivíduos, integrando, também, profissionais que precisam seguir se aperfeiçoando em busca de uma assistência qualificada e sustentável^(1,2)^.

Considera-se que todos os neonatos em uso de ventilação mecânica ou com dor avaliada deveriam receber analgésicos^(7,10)^. Apesar do alto número de intervenções farmacológicas deste estudo (Tabela 3), ainda há um caminho a ser percorrido. Os profissionais possuem receios no uso de medicações em neonatos, pelo metabolismo hepático e filtração glomerular serem reduzidos^(6,10)^, o que abre portas para o crescimento de intervenções não farmacológicas que, neste público, diferente das crianças, podem tê-las como adjuvante para o alívio álgico^(15)^.

Diante das competências essenciais de enfermeiros neonatais, a identificação de soluções alternativas para a promoção de saúde do neonato é estimulada^(15)^ e, somada ao 3º ODS, em busca de promoção do bem-estar^(1)^, as intervenções não farmacológicas devem ser ressaltadas. Essas possuem mecanismos de ação que enfocam na prevenção da desorganização, com efeitos neurológicos e endócrinos, sendo recomendado seu uso entre cinco e dez minutos antes e/ou durante a realização de procedimentos dolorosos^(6,7)^. Mas neste estudo, nota-se a baixa documentação dessas intervenções, principalmente as mais difundidas, como a solução adocicada^(15)^. Esse aspecto pode estar associado a dois pontos: receio ou rotina profissional.

Em investigação transversal com profissionais de saúde de unidades neonatais, notou-se que médicos e enfermeiros relatam receios de uso de intervenções não farmacológicas na prática clínica, com medo de repercussões na saúde do neonato, considerando sua labilidade clínica^(12)^. Reitera-se que esse receio não é algo negativo, porém o empoderamento científico é importante para a transição de receio à segurança (3º ODS). As intervenções não farmacológicas não estão livres de risco e devem ser bem utilizadas, como medidas de sucção, que não devem ser utilizadas em neonatos com anormalidades neurológicas e engasgos transitórios (não nutritiva), e anormalidades do trato gastrointestinal (nutritiva)^(8)^.

Outro ponto pode estar interligado à rotina profissional, que possui foco em fornecer um ambiente neutro, livre de estímulos, para conservação do consumo de oxigênio, da temperatura corporal e do metabolismo, com o manejo da dor e sua documentação ficando em segundo plano^(9)^. Aqui, houve 132 registros de redução da luminosidade, intervenção ambiental que corrobora o citado. A documentação da amamentação pode estar alta, considerando as políticas institucionais de hospitais amigos da criança, que visam ao estímulo à amamentação ao longo de toda a hospitalização de neonatos, condizentes com o 2° ODS^(1)^ e com o segundo eixo da PNAISC^(13)^.

Neste estudo, observou-se que o número de procedimentos dolorosos documentados foi elevado, como no caso de 70 punções arteriais, 32 acessos venosos, seis punções lombares e quatro flebotomias. Essa estimativa pode estar subestimada, e os profissionais com frequência documentam apenas o sucesso do procedimento, não o número de tentativas falhas. Em punções venosas, por exemplo, o número de tentativas pode ser elevado, considerando a rede venosa em desenvolvimento. Outro ponto é que esses procedimentos são de vital importância para a manutenção da vida do neonato. Expor o número de tentativas não é uma crítica à sua realização, mas sim a necessidade de refletir estratégias para que o neonato tenha uma redução do sofrimento durante o procedimento, indo, novamente, no caminho em direção ao bem-estar neonatal (3° ODS)^(1)^.

Existem outros procedimentos da rotina de cuidado que, apesar de irem contra a definição de procedimento doloroso^(12)^, podem causar dor se não forem bem conduzidos, como os cuidados de higiene, avaliação antropométrica e triagem neonatal, que devem ser refletidos em estudos futuros.

Torna-se premente a difusão das intervenções não farmacológica aos profissionais, como preconizado no 17° ODS (difusão de tecnologias). Contudo, é emergente que as intervenções sejam denominadas como tal, caminhando a uma cultura onde elas são vistas e valorizadas como uma tecnologia de cuidado, assim como as tecnologias duras, e transitem de opções a obrigações profissionais, integrando protocolos que visem ao bem-estar em UTIN. Neste estudo, observam-se oito intervenções não farmacológicas (com efetividade comprovada na literatura)^(6,19-22)^. Contudo, uma diretriz brasileira recente reconheceu apenas a sucção (nutritiva, não nutritiva e com soluções adocicadas), o contato pele a pele e a contenção facilitada^(8)^, demonstrando que essa reflexão também deve seguir a literatura científica.

Entre as inúmeras associações conduzidas, o pré-natal e o tempo de hospitalização foram associados ao uso de intervenções não farmacológicas. Diante do pré-natal, nota-se que 24,1% das mães não o realizaram (pré-natal negligente), fato preocupante e que vai contra o preconizado pelo 5° ODS (empoderamento de mulheres)^(1)^. Esse baixo número pode estar associado à pandemia de COVID-19, que impôs mudanças contextuais no sistema de saúde, já sendo reconhecido na literatura o baixo número de consultas no período^(23)^. Ainda, o pré-natal de qualidade é um estímulo inicial à parentalidade positiva^(10)^, e neste estudo, sua presença esteve associada ao estímulo à amamentação, o que pode ser associado a maiores orientações e preparo materno durante esse acompanhamento (2° e 5° ODS)^(1)^.

Já o pré-natal negligente esteve associado ao uso de intervenções não farmacológicas, aspecto positivo. Uma hipótese para esse achado é que essa variável pode ter estimulado o profissional a conduzir intervenções visando ao bem-estar do neonato (3° ODS)^(1)^. Outra possibilidade é o hospital coparticipante ser um hospital amigo da criança, que integra o Método Canguru e o aleitamento materno como partes integrantes do cuidado, garantindo ao neonato sua interligação aos dois eixos da PNAISC^(13)^. Essas intervenções foram as mais prevalentes no alívio da dor, resultado que pode corroborar o aspecto supracitado.

Há intervenções não farmacológicas que dependem da colaboração da família, como o estímulo à amamentação, o contato pele a pele/Método Canguru e a massagem terapêutica (onde se preconiza a condução pela família, supervisionada pelo profissional)^(12,22,24)^. Sua realização pode ser um preditor para a vinculação entre o binômio, proporcionando não só o bem-estar infantil, mas também o bem-estar materno (3° e 5° ODS)^(1)^. A UTIN pode ser um ambiente que impõe inúmeros desafios à saúde da mulher, somado às alterações hormonais após o parto, que podem prejudicar o vínculo^(9)^.

O vínculo entre o binômio é crucial para o desenvolvimento neurofisiológico, físico e psicoemocional do neonato. Contudo, a pandemia de COVID-19 influenciou este processo, como visto em estudo transversal com 127 binômios, em que observou-se que a gravidez não planejada e prováveis problemas de saúde mental materno prejudicaram a vinculação^(23)^. O 3° e o 5° ODS se integram novamente a esse processo, sendo necessário um olhar à saúde da mulher no contexto da UTIN^(1)^. Apesar do exposto, o número de contato pele a pele/Método Canguru deste estudo foi elevado. Todavia, ressalta-se que todos os neonatos deveriam recebê-lo, pois, para além do alívio álgico, é um estímulo à parentalidade positiva, à formação de vínculo, à promoção do bem-estar do neonato e uma intervenção de baixo custo, sendo sustentável e que deve ser incentivado na UTIN, indo de acordo com a assistência humanizada, eixo da PNAISC, e com o 3° ODS^(1,13,25)^.

Durante o período de vulnerabilidade neurobiológica, os neonatos podem passar por hospitalizações prolongadas em UTIN^(10)^, e este estudo demonstrou a associação dessa variável ao uso de intervenções não farmacológicas. Os pesquisadores indicam como hipótese para essa associação o aumento da chance de, no mínimo, uma documentação das intervenções, considerando que esse neonato será exposto a maior número de procedimentos dolorosos e um profissional pode fazer uma documentação. Por outro lado, cabe refletir que, na prática clínica, com o passar do tempo de hospitalização, os neonatos refinam os nociceptores e os mecanismos de organização à dor^(18)^, e isso pode fazer com que o profissional caia no comodismo em um cuidado mecânico: “Todo dia fazemos esse procedimento”^(26)^, sendo relevante ressaltar que a promoção do bem-estar é um processo contínuo que deve guiar toda a hospitalização^(1)^.

Ponto importante é que as intervenções não farmacológicas foram documentadas por enfermeiros, médicos e fisioterapeutas, o que demonstra que a educação (4° ODS) deve ser interdisciplinar, incluindo todos os profissionais que realizem procedimentos dolorosos^(1,7)^. Outro ponto é que este estudo foi conduzido no Brasil; em outros países, com outras culturas, as intervenções podem ter outra representação, especialmente as não farmacológicas, e outra disponibilidade. O cuidado culturalmente responsivo se torna uma filosofia que deve guiar também o manejo da dor, respeitando o contexto inserido, meta do 1° e 10° ODS^(1)^.

Refletir sobre o manejo da dor em UTIN com os ODS é caminhar em direção à prevenção de impactos a longo prazo no bem-estar materno-infantil. Apesar de o estudo fazer um recorte a uma etapa da vida de um indivíduo, é necessário ir além, uma vez que as experiências são acumulativas. A vivência de dor não aliviada pode levar a um efeito em cascata que prejudicará a criança, o adolescente, o adulto e o futuro idoso^(2,8)^. O manejo da dor em neonatos é um cuidado para o presente e para o futuro, estando de total acordo com a premissa da Agenda 2030 para o Desenvolvimento Sustentável^(1)^.

Após a hospitalização, esse neonato será cuidado em uma Rede de Atenção à Saúde, que deve estar interligada com ações em um território, para a promoção do desenvolvimento pleno do indivíduo. É necessário articular ações que respeitem o contexto social (1° e 10° ODS) e promovam a segurança alimentar (2° ODS), educação de qualidade (4° ODS), saneamento básico e respeito ao meio ambiente (6°, 13°, 14°, 15° ODS), acesso a tecnologias sustentáveis (7°, 8°, 9°, 11° ODS) e universal aos serviços de saúde (16° ODS), que tenham o desenvolvimento sustentável como guia para articular o cuidado (17° ODS)^(1-2)^. O manejo da dor em UTIN é um pequeno recorte temporal de toda a importância de se articular um cuidado em busca do bem-estar (3° ODS)^(1-2)^, contudo é tão importante quanto os supracitados.

### Limitações do estudo

Este estudo apresenta como limitações seu recorte retrospectivo, o que leva a uma perda de informações que seriam essenciais para uma análise mais aprofundada do fenômeno, como o número de procedimentos dolorosos realizados, incluindo as tentativas, e a avaliação da dor realizada por parâmetros subjetivos, o que pode ter subestimado a vivência álgica real.

### Contribuições para a área da saúde, enfermagem ou políticas públicas

Em um mundo que caminha para o desenvolvimento sustentável do indivíduo, o manejo da dor neonatal se torna crucial para os profissionais de saúde, com ênfase no enfermeiro que atua no gerenciamento da dor. Este estudo demonstrou um número elevado de procedimentos dolorosos, mas com baixo número de documentações da dor e intervenções. Esse aspecto pode representar uma prática clínica que precisa ser ressignificada, caminhando para a integração dos ODS em um cuidado seguro e de qualidade à saúde do neonato, reduzindo as repercussões da vivência álgica não aliviada a longo prazo. Os dados deste estudo e as reflexões discutidas podem ser um pontapé inicial para esse longo caminhar.

## CONCLUSÕES

Neste estudo, observou-se que o manejo da dor em neonatos foi desvalorizado. Todos os participantes foram expostos a, no mínimo, um procedimento doloroso, contudo apenas 13,7% tiveram documentação de dor, sem uso de escala validada, apesar de disponível. Houve uso de intervenções farmacológicas, como fentanil contínuo ou intermitente, e intervenções não farmacológicas, prevalecendo o estímulo à amamentação e o Método Canguru, com associação dessas intervenções à realização do pré-natal e hospitalização prolongada (p<0,05). Apenas 2,8% dos neonatos foram reavaliados. Esse contexto se interliga aos ODS, com enfoque no bem-estar, considerando que a vivência álgica pode repercutir ao longo do desenvolvimento, sendo relevante que o manejo da dor se integre às metas preconizadas para o desenvolvimento sustentável.

Para o avanço na saúde global de neonatos, recomendamos a necessidade de: formulação de estudos de tradução de conhecimento que objetivem implementar as tecnologias de cuidado para alívio da dor, desenvolvimento do neonato e promoção do bem-estar na prática clínica; a interligação do contexto social, econômico e cultural da família nas estratégias de difusão e em um cuidado responsivo; o financiamento de estudos relacionados a neonatos e ODS por agências de fomento; a educação interprofissional, por meio de estratégias inovadoras que permitam a difusão de tecnologias e empoderamento científico; um olhar de profissionais à saúde da mulher, do neonato e da criança, desde o cuidado pré-natal, com uma interligação da Rede de Atenção à Saúde em um cuidado territorial.
